# Recommendations for selection and adaptation of rating scales for clinical studies of rapid-acting antidepressants

**DOI:** 10.3389/fpsyt.2023.1135828

**Published:** 2023-06-02

**Authors:** Christian Yavorsky, Elizabeth Ballard, Mark Opler, Jan Sedway, Steven D. Targum, William Lenderking

**Affiliations:** ^1^Valis Biosciences, Inc., Berkeley, CA, United States; ^2^NIH/NIMH, Bethesda, MD, United States; ^3^WIRB Copernicus Group (WCG) Clinical Endpoint Solutions, Princeton, NJ, United States; ^4^Signant Health, Blue Bell, PA, United States; ^5^Evidera, Bethesda, MD, United States

**Keywords:** rapid acting anti-depressants, psychometrics, measurement of rapid response to anti-depressant treatment, ISCTM working group, novel anti-depressants, ketamine, psychedelics

## Abstract

The novel mechanisms of action (MOA) derived from some recently introduced molecular targets have led to regulatory approvals for rapid acting antidepressants (RAADs) that can generate responses within hours or days, rather than weeks or months. These novel targets include the N-methyl-D-glutamate receptor antagonist ketamine, along with its enantiomers and various derivatives, and the allosteric modulators of gamma-aminobutyric acid (GABA) receptors. There has also been a strong resurgence in interest in psychedelic compounds that impact a range of receptor sites including D1, 5-HT7, KOR, 5-HT5A, Sigma-1, NMDA, and BDNF. The RAADs developed from these novel targets have enabled successful treatment for difficult to treat depressed individuals and has generated a new wave of innovation in research and treatment. Despite the advances in the neurobiology and clinical treatment of mood disorders, we are still using rating instruments that were created decades ago for drugs from a different era (e.g., The Hamilton and Montgomery-Åsberg depression rating scales, HDRS, and MADRS) continue to be used. These rating instruments were designed to assess mood symptoms over a 7-day time frame. Consequently, the use of these rating instruments often requires modifications to address items that cannot be assessed in short time frames, such as the sleep and appetite items. This review describes the adaptative approaches that have been made with the existing scales to meet this need and examines additional domains such as daily activities, side effects, suicidal ideation and behavior, and role functioning. Recommendations for future studies are described, including the challenges related to implementation of these adapted measures and approaches to mitigation.

## Introduction

Until 2019, approved antidepressant treatments targeted the biogenic amines and were based on assumed mechanisms of action (MOA) that had been the mainstay of drug development and the psychiatric treatment of mood disorders for decades. However, in March 2019 the regulatory approval of two new treatments (esketamine approved for treatment resistant depression and brexanolone approved for post-partum depression) moved the field beyond the MOA of prior decades (1). With new synaptic targets, such as the N-methyl-D-glutamate receptor antagonist (NMDA) and gamma-aminobutyric acid (GABA), the core assumptions about what antidepressants are and how they work have been challenged and the course of drug development in psychiatry has been altered.

One important distinction about these new treatments and conventional antidepressants is the speed with which they begin to ameliorate depressive symptoms. Standard antidepressant treatments, acting on dopamine, norepinephrine, and/or serotonin take several weeks to work, and the course of treatment may take many months. Additionally, 30–40% of depressed individuals are resistant to conventional SSRI/SNRI treatments and results from large population studies suggest that 65% of treated individuals will relapse within 3 months (2, 3). Alternatively, the rapid acting antidepressants (RAADs) have the potential to resolve symptoms in a much shorter timeframe. For instance, Opler et al. (4) described rapid positive responses to administration of 40 mg of intranasal ketamine under supervision after only 15 min. The pattern of symptom response may differ somewhat from the responses to the traditional, more conventional treatments. In addition to the reduction in sadness and anxiety, investigators have noted an ultrarapid transition from pessimism to a markedly improved outlook that includes the use of positive valence in language and the onset of what one treated individual described as “calm alertness.” This notion of “calm alertness” also appears to be an area for further development and discussion: are the changes seen in the symptom severities measured by these instruments the most salient for patients? Scales such as the Clinician Administered Dissociative States Scale (CADSS) (5), 5-Dimensional Altered States of Consciousness Rating Scale (5D-ASC) (4, 6, 7), Mystical Experience Questionnaire (MEQ) (8, 9), and others measure aspects of subjective experience important to treatment effect are often included in clinical trials. That said, they are often not included in primary analysis and exist as secondary outcome measures to asses risk. The literature around treatment response to RAADs and psychedelics in terms of these types of scales suggest strong correlations between domains like insight, feelings of unity and empathogenic effects [e.g., (10–13)] and antidepressant treatment response. Notably, Roseman et al. (13) found correlations of “oceanic boundlessness” (*r* = 44) on the 5D-ASC and reduction of depression severity as measured by the self-reported QIDS-16 and was also predictive of treatment response (based on a clinical response defined as ≥50% in QIDS-16SR at 5 weeks compared to baseline). Griffiths et al. (14) also explored immediate and persistent positive experiences that overlap somewhat with typical depression rating scales but capture phenomenon such as the above mentioned “unity” as well as “deeply felt positive mood” and positive attitudes toward self, life, and others. While not a focus of the working group at his time, the question of whether the current scales are measuring patient experience in a thorough and representative manner are critical and merit further discussion.

Prior eras of clinical trials saw innovation in psychiatry spur a set of new approaches to evaluation and assessment of efficacy and safety. Drug treatments for depression led Dr. Max Hamilton to create a new, clinician-rated scale that was specific to patients “suffering from affective disorder of the depressive type” (15). Hamilton developed the Hamilton Depression rating scale (HDRS) to facilitate communication between physicians and not for the serial assessment of mood symptoms in clinical trials. Twenty years later, a profusion of clinical trials for new candidate drugs required a more sensitive, broadly applicable rating instrument that would be capable not only of differentiating drug from placebo but assessing entirely different classes of treatment as well. Consequently, the Montgomery-Åsberg depression rating scale (MADRS) was developed by Montgomery and Åsberg (16) in a validation study. These investigators created a 10-item rating instrument that selected the 10 symptoms that were most sensitive to change during a clinical trial of antidepressants that were available at that time. Subsequently, new measurement tools flourished, including patient reported outcomes, such as the Beck Depression Inventory (1961). The HDRS and MADRS, further operationalized by Williams' (17) and the SIGMA by Williams and Kobak (18) formalized the use of structured interview guides, the practice of euthymic baseline comparisons, and helped standardize the past week as the reference period for depression assessments in clinical research. The use of functional assessment tools such as the Sheehan Disability Scale (SDS) with its DISCAN metric (19, 20), and the advent of new safety measures to capture suicidality, including the C-SSRS (21), S-STS (22), and others further established the core set of assessments which appeared to be sufficient, although concerns about the “gold standard” have been raised periodically [e.g., (23, 24)]. It is important to note that none of these instruments were developed for the assessments of RAADs.

The experience of the “first generation” of RAAD development has yielded some important lessons, principally about the best practices in the use of these existing scales. Under the auspices of the International Society of CNS Clinical Trials Methodology (ISCTM), a working group co-chaired by four of the authors (CY, EB, MO, and JS) was convened to evaluate the approaches undertaken to date. In the following sections, we will discuss some of the efforts made to adapt the existing scales for RAAD studies, the changes that have been made in each, and the evidence, if any for the effectiveness of those adaptations. Additionally, we will outline the development of new scales and the possibilities that may be afforded by novel digital measurements.

## Defining the “RAPID-acting” paradigm: when is “rapid” and what is “action?”

The ISCTM working-group noted that “rapid-acting” is a challenging term to define and that there has been a lack of consensus in the field on the most appropriate criteria. Broadly speaking, “rapid-acting” may be taken to mean any measurable, significant improvement that manifests in shorter timeframes than those known to be associated with standard treatments. Gould et al. (25) indicate that “ideally, a rapid-acting antidepressant drug would exert its effects within hours or days, as opposed to weeks or months.” Standard evaluations in antidepressant trials have typically been conducted at weekly in-clinic visits. Clinicians are asked to summarize a week's experience of symptoms into a narrow quantitative measure despite the fact that symptom severity may vary from day to day. Nonetheless, the one-week time frame has been largely effective for the evaluation of conventional antidepressants because the clinical trials lasted for 4–8 weeks. However, the experience of clinicians from the past 60 years would suggest that detectable, meaningful symptomatic changes may occur in <1 week and that the 1-week time frame may be insufficient for new drug candidates. The emergence of RAADs have convinced everyone that shorter measurement intervals are needed. As noted above, results from studies of ketamine, arguably the archetype of this class of treatments, have shown early responses that are clinically meaningful to affected individuals. These findings have built a case for evaluative methods that are closer to real-time.

The advent of RAADs raises some unresolved questions about the criteria for clinically meaningful response. It is fairly well-understood that many RAADs like ketamine may have an early impact only certain domains of depressive symptoms [e.g., (26)], that some symptoms cannot be immediately measured (sleep, appetite), and that the duration of effect can be a week or less [e.g., (27)]. Hence, there are open questions about the requisite number of symptoms and the length of symptom resolution needed to call a treatment an antidepressant:

1. How many depressive symptoms need to resolve in order to describe a response as a clinically meaningful antidepressant response?
2. How long do the resolution of symptoms need to last in order to describe a response as a clinically meaningful antidepressant response?

The first question is compounded by the reality that not all depressed individuals have the same depressive symptoms. The second question is compounded by the issue of dosing frequency.

Defining what constitutes a clinically “significant” effect for RAADs seems simpler in some respects than defining what is clinically meaningful because there are well-established standards for statistical and clinical significance that have been used for many years by methodologists and regulators to evaluate evidence from clinical trials. Often, the threshold for antidepressant response has been ≥50% decrease from the baseline MADRS or HDRS score [e.g., (28)] whenever that occurs.

An ongoing debate within our workgroup reflects the dynamic nature of this concept of response as it applies to RAADs, specifically: should change be taken to mean rapid alleviation of symptoms that reach or approach statistical significance or should it require rapid remittal of symptoms below some pre-defined threshold? A further challenge, as noted in question 2 above is the need to demonstrate not merely early improvement, but also sustained relief. Is rapid, transient improvement acceptable or useful, given the risks associated with starting any new treatment with a patient who suffers with a mood disorder? Furthermore, the heterogeneity of symptom resolution also requires consideration; for example, improvements in subjective mood without functional improvements or reduction in suicidal ideation would generally not be considered adequate by most stakeholders. As these topics are still open for debate, we must be precise about the timeframe of assessment, the maintenance of response, and the domain under study in any discussion about the merits or limitations of RAADs.

In the conduct of this review, we have primarily focused on documenting the methods used for evaluation, the approaches taken to date in modifying standard assessments, and the evidence for their effectiveness. This work focuses on how investigators have chosen to alter rating scales for use in rapid-acting antidepressant studies, as well as the evidence for continued reliability and validity. We have included several of the major clinician-rated measures routinely used as primary efficacy endpoints, as well as the most widely utilized tools to evaluate suicidal ideation and behavior, functional status, quality of life, and side effects common to some classes of molecules, and other aspects of safety and efficacy.

Over the course of the working group's activity a number of alternatives were proposed and developed. In general terms these were:

- the adaptation of existing scales or measurement techniques
- the development of new scales
- and the inclusion of digital measurements to supplement existing scales.

## Measuring symptom severity and efficacy of RAADs with existing rating instruments

### MADRS/SIGMA

Singh et al. (29) used both full and abbreviated MADRS scores in the assessment of response to IV esketamine. They assessed depressive symptoms at 2, 4, and 24 h following the infusion and used the term “since the last assessment” for the 2 and 4-h periods where the sleep and appetite items were omitted. In Johnson et al. (30) they evaluated the suitability of the MADRS within a 24-h recall period as opposed to the standard reference period indicated above as the past week and found that this had equal content validity with high internal consistency reliability (Cronbach α of 0.84 and 0.91) and test-retest reliability (intraclass correlation coefficients of 0.96 and 0.91). According to their study “the majority of participants reported that a meaningful change in depression symptoms could be assessed in a 24-h recall period, except for reduced sleep and appetite.” This echoes Singh et al. (29) and their decision to omit these items for the shorter recall period. Using esketamine trial data, Yavorsky et al. (31), noted that the MADRS was sensitive to change across short periods though the sleep and suicide item did not appear to show significant sensitivity to change reinforcing the idea that rapid change is most often associated with mood symptoms. The conventional triad of depressive symptoms (mood, appetite, and sleep disturbances) does not apply neatly into a 24-h time frame for assessment of clinical change.

### HDRS/SIGH-D

Bunney and Bunney (32), while investigating the rapid-acting antidepressant effect of ketamine paired with sleep modification, noted significant decreases in HAMD scores within a 48-h period using an unmodified version of the scale (and indicated that many patients met criteria for remission in this period). Their paper also provides a meaningful review of the literature around time-to-response for a range of standard antidepressant compounds. Luckenbaugh et al. (33) compared the use of the 17 item HDRS, and 8 other shortened versions of the HDRS, including one or two item measures within ketamine clinical trials. Overall, the one or two item scales demonstrated the smallest effect sizes to ketamine, while the 17-item HDRS was associated with the lowest response rates. They also noted that the “response rates for HDRS total score were the smallest of all the scales, including those for single item measures. This is most likely due to the fact that a few items are included in the total score that cannot change measurably over the brief time frame.” This again suggests the sensitivity of more durable symptoms may be limited while the alleviation of primary mood symptoms appears robust and measureable with these types of rapid-acting compounds. Milak et al. (34) employed a similar rationale in their use of the HDRS-24 that excluded those items for diurnal variation and loss of weight in their study examining the impact of ketamine on glutamate, glutamine (Glx) and γ-aminobutyric acid (GABA) level and symptoms of depression.

### HAMD6

The six-item version of the HDRS was developed by Per Beck and is a valid and sensitive scale for assessing depressive symptoms in clinical trials (35, 36). The HAMD6 can be used for RAAD studies because it is designed to query symptoms experienced in the past 72 h and can be modified for shorter durations as well. The 6 items include depressed mood, loss of self-esteem, loss in interest, psychomotor retardation, psychic anxiety, and somatic symptoms. The HAMD6 does not inquire about sleep or appetite. In two clinical trials of RAADs, HAMD6 scores improved significantly more than placebo assigned subjects (37, 38). In one exploratory study of a potential RAAD (an mTORC1 activator), the candidate drug was significantly better than placebo on the HAMD6 at 4 and 12 h after dosing (37).

### IDS/QIDS-SR16

The abbreviated form of the Quick Inventory of Depressive Symptoms (QIDS-SR16) by Rush et al. is a 16-item patient-rated instrument that has been shown to be sensitive change in patients undergoing RAAD treatment (39, 40). In both studies, participating patients completed the QIDS-SR16 ~2 days after each IV ketamine infusion.

Lenderking et al. (41) had previously developed the QIDS-SRD14 as a daily measure of depressive symptoms for the purpose of detecting rapid treatment effects. The scale takes the 16 items of the QIDS and eliminates the items pertaining to sleep and weight, rewording the other items to be relevant in a 24-h response window. The scale was used in a depression methods study and showed significantly faster detection of treatment onset and response than the HAM-D or MADRS administered weekly (42).

### PHQ-9

The PHQ-9 is useful for screening individuals for depression (43). The PHQ-9 guidelines indicate that the symptoms should be measured over the “past 2 weeks” and in many studies [e.g., (44)], the instrument was used in this manner. Used in this manner, the PHQ-9 has shown sensitivity to change in response to RAAD treatment in an esketamine study [e.g., (45)]. The changes noted across the PHQ-9, MADRS and CGI-S in this study showed consistency in change scores from baseline to Day 15 and 28. While, Artin et al. (46) also reported similar findings with the PHQ-9 in terms of detection of change over time, we did not locate any studies that used a modified recall period for this instrument.

### BDI

The Beck Depression Inventory (BDI)I has been shown to be sensitive to change in general in the treatment of depressed individuals. Wilkinson et al. (47), noted that the BDI was uniquely insensitive to suicidality (when compared to the HAMD, MADRS and QIDS-SR). Nonetheless, other studies [e.g., (48)] used it as a treatment outcome measure to assess changes over a 24-h period in response to ketamine treatment for MDD. Weigand et al. showed a significant change in BDI score from baseline was 34.1 (*SD* = 11.3) to 24.7 (*SD* = 10.0) 24 h after ketamine administration [*t*_(1, 23)_ = 4.1, *P* < 0.001].

## Consideration of the adaptation of existing scales for RAAD trials

While several of the studies cited above employed abbreviated or otherwise modified versions of existing scales, there were none that specifically validated the versions that were used in their studies. The RAAD working group agreed that, ideally, for an appropriate psychometric validation of the modified HAMD and MADRS versions that have excluded sleep and appetite, an item-response theory (IRT) analysis should be conducted to determine the item-level contribution when used with these populations. Insofar as techniques that could be used for the development of additional scale in the investigation of rapid-response, the “discan” (discretized analog) method was suggested.^1^ This method allows for a more clinically oriented categorization and, according to Singh and Bilsbury (49), it can be used for “obtaining reliable and precise measures of subjectively experienced dysfunction variables whose possible values form a continuum.”

Discretization is a term borrowed from applied mathematics and, in this context, translates to breaking down an essential concept (e.g., a continuous variable) into discrete (measurable) items that are familiar to psychometricians. While this method has been in practice since the mid-1980s and provides a more organized and replicable way to generate new scales, it has not yet been applied to the problem of measuring rapid acting anti-depressants and needs further research and attention.

Alternatively, the working group recognized that the HAMD-6 is already a well-validated instrument but noted that has had limited use in RAAD trials to date.

## The development of new scales

The development of novel scales for the assessment of RAADs and other antidepressants has been underway. Sonya Eremenco (Executive Director of the Patient-Reported Outcome Consortium at The Critical Path Institute) presented her research to the ISCTM RAAD working group on a newly validated scale called the Symptoms of Major Depressive Disorder Scale (SMDDS) (see Appendix 1 for conceptual framework) that was developed in conjunction with the FDA and Critical Path Institute (C-PATH). This scale follows the work of McCarrier et al. (50) and Bushnell et al. (51) in which they outlined their considerations of FDA guidance around best practices as well as the development of a patient-reported outcome with high content validity. According to FDA documents (52) [Clinical Outcome Assessments (COA) Qualification Program DDT COA #000109]: “The intent is to use the SMDDS to assess treatment benefit in clinical trials for MDD therapies that may have a faster onset of action and potentially communicate this earlier antidepressant treatment benefit in the product label.” At this writing, the scale has not been used in a clinical trial with rapid-acting antidepressants but given its sensitivity to change over shorter time durations and specific construction for this purpose, it may play a role in studies using rapid-acting antidepressants moving forward.

McIntyre et al. (39, 53) developed the McIntyre And Rosenblat Rapid Response Scale (MARRRS) with the specific aim of measuring rapid treatment response. The MARRRS is a self-report measure that contains 14 items scored 0–4 based upon symptoms experienced in the past 72 h. The psychometric characteristics of the MARRRS appear very promising. In a validation study, the MARRRS had a high internal consistency across acute infusions as determined by Cronbach's alpha (0.84–0.94). There was significant convergent validity between the QIDS-SR-16 and MARRRS total scores across infusions [*rs*_(292)_ = 0.87, *p* < 0.001]; the MARRRS was also sensitive to change [*rs*_(49)_ = 0.70, *p* < 0.001]. It is noteworthy that an exploratory factor analysis showed that MARRRS items loaded onto two factors (i.e., dysphoria and psychic anxiety) accounting for 63.4% of the total variance.

This scale will be used in an upcoming Phase II trial (Psilocybin for Treatment-Resistant Depression: NCT05029466). Gukasyan et al. (54) indicate that psilocybin “produces substantial and rapid antidepressant effects in patients with major depressive disorder (MDD).”

Based on the ISCTM working group's consensus, the areas of depressive illness that appear to be most apt to change in short periods of time (e.g., depressed mood, anxiety, suicidal ideation, and anergia) mirror those items validated in the MARRRS.

## The inclusion of digital methods to supplement existing scales

The working group expressed a great deal of interest in the exploration of digital measurements to assess RAADs, but the consensus agreed no single system was ready to be used as a primary tool in a clinical trial. Digital methods have the advantage of periodic clinician assessments by collecting moment to moment reports that might be more accurate because they are essentially “real time” measures obtained directly from the source (the participant). There have been significant advances in the use of digital methods as it applies to the measurement of depressive symptoms [e.g., (55–57)]. Onnela et al. demonstrated the utility of smartphone-based applications that allow for more ecologically valid assessment of depressive symptoms over time and in an Aledavood et al. (58) review, the prevalence and validity of a range of techniques for measuring symptoms using actigraphy and other features that are built into many smartphone platforms. To paraphrase Kamath et al. (59), non-standardized assessments that use ecological momentary assessments (EMA) and other digital phenotyping techniques usually lack validation but may provide important clinical information that is not generally accessible in the context of a study or clinic visit. Validation of EMA applications has begun in clinical trials for depression. In a open label antidepressant trial, Targum et al. (60) found consistent correlations between clinician reported outcome measures (HAMD-17 and HAMD-6) and an EMA derived HAMD-6 completed by the participating subject using a smartphone. They noted that the daily EMA reports anticipated clinical outcome before the clinician visits and demonstrated consistent symptomatic improvement or little clinical change from day to day. The use of EMA pre-randomization to establish critical baseline stability was suggested as essential in the study of RAADs.^2^ This baseline stability is critical because EMA provides a “snapshot” of behavior that may not be the most accurate assessment of a patient's overall symptom severity, thus a demonstration of stability by multiple EMA entries will be important in the ability of researchers to assess the impact of a rapid-acting compound over time.

With advances in machine learning (ML) to analyze facial and vocal data [e.g., (61)] and the near ubiquity of smartphones to provide passive data there will no doubt be integration of these methods into upcoming trials in this space.

## Conclusion

While this review shows that with the described adaptations, existing standard rating instruments can capture some symptomatic change in the timeframe in which RAADs act, they remain inherently limited by their structure (items like sleep and appetite), timeframe of retrospective review of symptoms (usually 7 days) and conceptual biases. It is also noteworthy that, to date, FDA has accepted only clinician rated outcome measures (HAMD, MADRS, and CDRS-R) in Phase III trials as primary endpoints to support an indication for mood disorders. All the traditional rating instruments described in this review have strong literature support for their validity, reliability and sensitivity to change, but these instruments have principally been conceptualized and tested under specific, older treatment paradigms (e.g., MAOIs, TCAs, and SSRIs). Despite past successes, the ISCTM working group concluded that it is likely that these measures fail to capture some aspects of the patient experience following treatment with RAADs.

Both the work of the Critical Path Institute and McIntyre et al. have shown that scales specific to the detection of change in depressive symptoms can be developed and have, in a preliminary way, been deployed into clinical trials. Given the psychometric properties and intent of their design for RAADs, both the SMDDS and MARRRS have significant promise.

Digital technologies including ecological momentary assessments (EMA), sleep and activity trackers as well as a range of applications that use facial and vocal metrics have been developed and should, initially, work to supplement and cross-check the items in existing or new scales that overlap. For EMA applications this may serve to provide more real-time assessments of the patient's mood states over the past week, while sleep and activity applications would seem to bolster or validate patient reports in these domains of depressive symptomology. Voice and facial metrics may be the closest to being sensitive to rapid changes in mood that can be reflected in affect or voice parameters.

Further development of new measures that meaningfully evaluate the novel effects of RAADs will require new approaches, as radical as the departure was from the older generations of drugs to the newer ones. The rapidity of effect that these new treatments exhibit may be the most obvious hallmarks of RAADs, but the speed with which they are reported to work might not be the feature which best distinguishes them. To ensure we are fully and objectively measuring what matters most to patients, clinicians and other stakeholders, our rating instruments and perhaps even our most basic assumptions about what they are and how they work may also have to adapt to keep up with the remarkable pace of change in the field of RAADs.

## Author contributions

CY, EB, MO, JS, ST, and WL participated in the Rapid-Acting Anti-Depressants working group and in the research, compilation, and writing and editing of this manuscript. Additional credit is given to the ISCTM RAAD Working Group at large who were able to provide a broad range of perspectives from regulatory to academic to industry. All authors contributed to the article and approved the submitted version.

## Footnotes

^1^Suggested by working group member David Sheehan, MD.

^2^Suggested by working group member Steve Targum, MD.
