# Supplementary figures and images for: Recommendations for selection and adaptation of rating scales for clinical studies of rapid-acting antidepressants
Source: Front Psychiatry. 2023 Jun 2;14:1135828. doi: 10.3389/fpsyt.2023.1135828 (PMC10272853; doi:10.3389/fpsyt.2023.1135828)

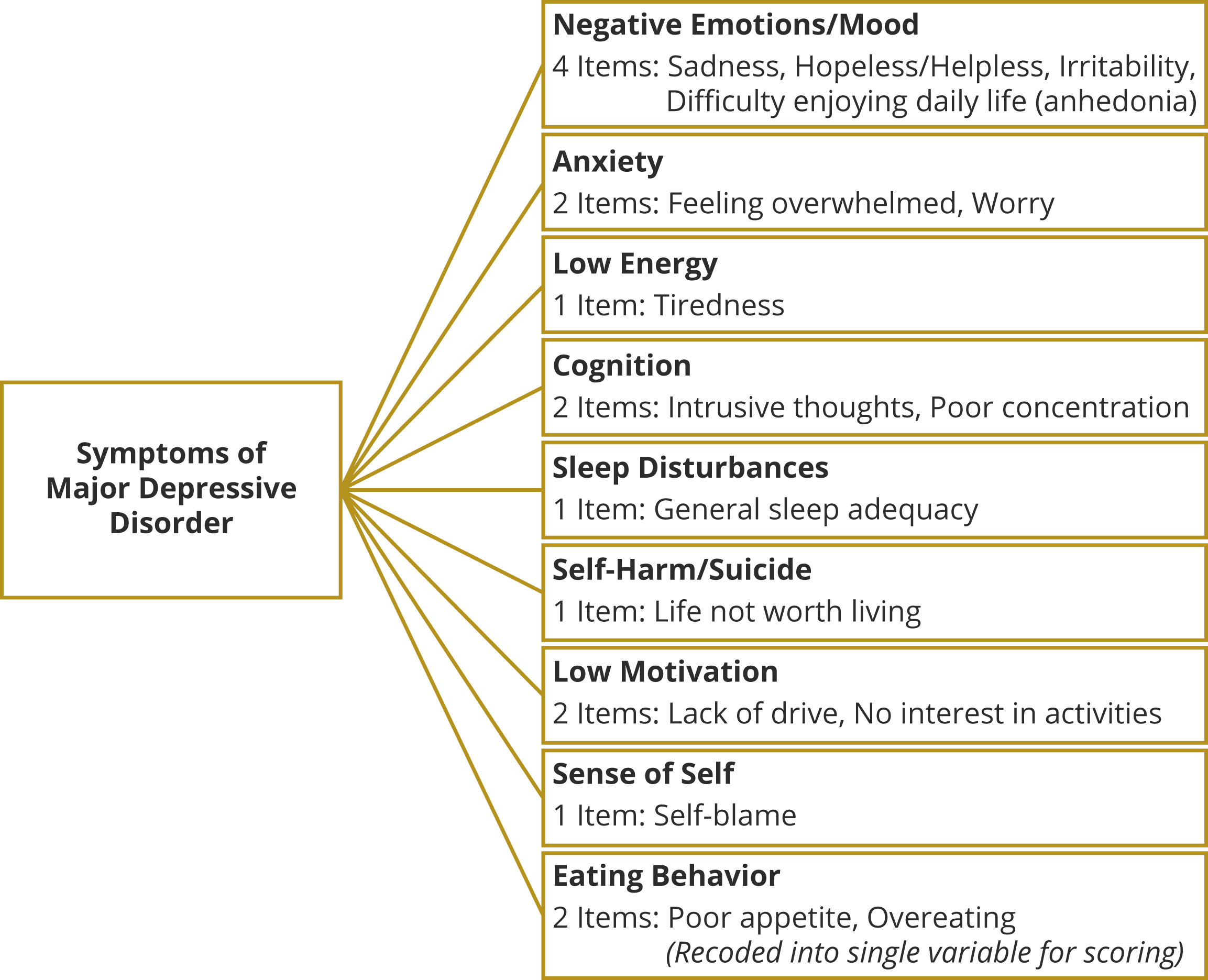

Supplement: Supplementary file 1 [file Image_1.jpeg]
